# Interconnection of CD133 Stem Cell Marker with Autophagy and Apoptosis in Colorectal Cancer

**DOI:** 10.3390/ijms252011201

**Published:** 2024-10-18

**Authors:** Ferenc Sipos, Györgyi Műzes

**Affiliations:** Immunology Division, Department of Internal Medicine and Hematology, Semmelweis University, 1088 Budapest, Hungary

**Keywords:** CD133, autophagy, apoptosis, colorectal cancer, stem cell

## Abstract

CD133 protein expression is observable in differentiated cells, stem cells, and progenitor cells within normal tissues, as well as in tumor tissues, including colorectal cancer cells. The CD133 protein is the predominant cell surface marker utilized to detect cancer cells exhibiting stem cell-like characteristics. CD133 alters common abnormal processes in colorectal cancer, such as the phosphoinositide 3-kinase (PI3K)/protein kinase B (AKT) and Wnt/β-catenin pathways. Autophagy is a cellular self-digestion mechanism that preserves the intracellular milieu and plays a dual regulatory role in cancer. In cancer cells, apoptosis is a critical cell death mechanism that can impede cancer progression. CD133 can modulate autophagy and apoptosis in colorectal cancer cells via several signaling pathways; hence, it is involved in the regulation of these intricate processes. This can be an explanation for why CD133 expression is associated with enhanced cellular self-renewal, migration, invasion, and survival under stress conditions in colorectal cancer. The purpose of this review article is to explain the complex relationship between the CD133 protein, apoptosis, and autophagy. We also want to highlight the possible ways that CD133-mediated autophagy may affect the apoptosis of colorectal cancer cells. Targeting the aforementioned mechanisms may have a significant therapeutic role in eliminating CD133-positive stem cell-phenotype colorectal cancer cells, which can be responsible for tumor recurrence.

## 1. Introduction

Nowadays, colorectal cancer (CRC) is the third most common cause of cancer death worldwide [1]. It is a sad fact that CRC has an increasing incidence and mortality in individuals under 50 years of age [2]. Early detection of the disease is essential for improving prognosis [3]. In addition to genetic mutations and epigenetic abnormalities, chronic inflammation may also play a role in the development of CRC, which together leads to malignant transformation of epithelial cells and subsequently increased tumor cell proliferation through an imbalance in cell division and cell death [2,4,5]. The complex processes of apoptosis and autophagy are important in almost all steps of CRC development and progression [6,7].

The intricate mechanism of apoptosis involves several upstream regulators and several downstream effectors [8]. The final “apoptotic trigger” is regulated by the balance between the pro- and anti-apoptotic functions of the regulatory protein components. In addition, a number of key molecules have been identified that play a key role in tumor development, most notably the DNA damage sensor acting through the tumor suppressor p53 (TP53) [9,10].

Autophagy is a cellular process intended to regulate protein expression levels, the turnover of damaged organelles, and the persistence of long-lived proteins in order to preserve cellular homeostasis. The role of autophagy in CRC is multifaceted, as it affects CRC development, progression, and even anticancer drug resistance, which determines the outcome of therapy [11,12].

Numerous functional connections link apoptosis and autophagy, sharing common signaling pathways. This is partly due to the fact that they have similar protein components. Simultaneous activation of the two machineries in the same cell during cancer development can sometimes interfere with the autophagy mechanism through apoptosis or inhibit apoptosis by autophagy [13].

Cancer stem cells (CSCs) were discovered in leukemia as early as the 19th century, but it is only since 1970 that CSCs have been intensively studied [14,15]. Most tumors contain CSCs, which are self-renewing cell types that are involved in tumor development, growth, resistance to treatment, recurrence, and metastasis formation [16]. The expression of cell surface markers that can also identify CSCs depends on tumor type [17]. CD133, a known CSC marker, has been shown to be a useful diagnostic marker for CRC, as CD133 positivity above 3% is strongly correlated with spheroid formation by cancer cells. This rate also radically reduces the survival of CRC patients [18,19,20]. In our own cell-free deoxyribonucleic acid (DNA) HT29 cell culture experiments investigating the Toll-like receptor (TLR)9-mediated autophagy response, we found that in some experimental set-ups, HT29 cell death is enhanced, while at the same time we identified a small number of surviving HT29 cells representing a CD133+ stem cell phenotype [21,22]. This could be the basis for disease recurrence. The aim of our current review is to summarize the effects of the CD133 molecule on apoptosis and autophagy in CRC, providing a reference for future research.

## 2. Structure and Function of CD133

**Figure 1:**
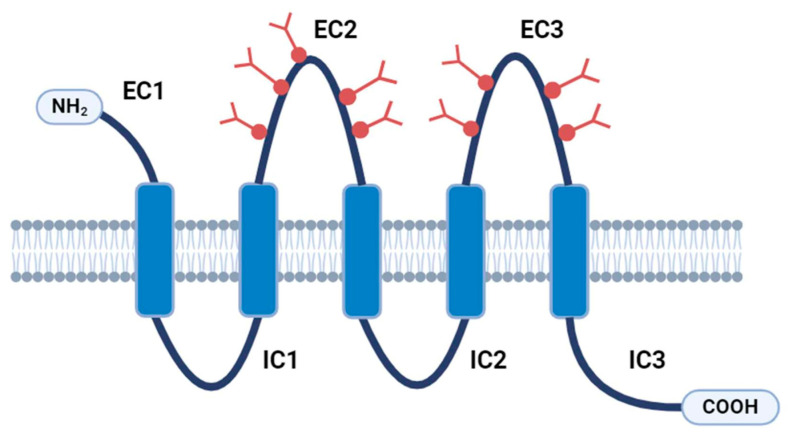
The structure of CD133. Five membrane-spanning domains make up CD133, with the N-terminal domain (NH2) exposed to the extracellular milieu, four alternating short cytoplasmic and large glycosylated (red symbols) extracellular loops, and a cytoplasmic C-terminal domain (COOH). A large number possess unique cytoplasmic C-terminal domains, which may suggest the presence of a variety of cytoplasmic protein-interacting partners. EC: extracellular domain; IC: intracellular domain. The figure was partially created with https://www.biorender.com (accessed on 20 September 2024).

CD133, a 97 kDa pentaspan transmembrane glycoprotein, comprises an extracellular N-terminal domain (EC1), five transmembrane segments that divide two short intracellular loops (IC1 and IC2), two larger extracellular loops (EC2 and EC3), and an intracellular C-terminal domain (IC3) [23]. The two extracellular loops contain nine putative N-glycosylation sites, with five located on the EC2 domain and four on the EC3 domain [24] (Figure 1). Glycosylation alters the general tertiary structure and stability of CD133, leading to the production of a 120 kDa protein [25]. *Prominin 1* (*PROM1*), the CD133 gene, is located on chromosome 4 in humans and chromosome 5 in mice. The homology between primates and rodents is only approximately 60% [25,26]. Human CD133 transcription occurs through five alternative promoters, three of which are located on CpG islands and partially regulated by methylation. These promoter regions frequently induce alternative splicing of CD133 messenger ribonucleic acid (mRNA), resulting in CD133 structural variants with potentially unique roles [27,28,29].

The physiological functions of CD133 in normal biological processes and its involvement in cancer progression are still not fully understood. Mostly found in plasma membrane protrusions and microvilli, CD133 plays a role in membrane organization [30,31]. CD133 is able to connect directly to lipid rafts that contain cholesterol, which enables its involvement in several signaling cascades [32]. CD133 knockout mouse observations support the assumption that CD133 acts as a scaffolding protein. Furthermore, other research has indicated that CD133 may play a significant role in selecting the destiny of cells or preserving their stem cell characteristics [33,34,35]. Contrarily, CD133 expression is not limited to intestinal stem or cancer-initiating cells; during the metastatic transition, CD133+ tumor cells may generate a more aggressive CD133- population, which can also initiate tumors in NOD/SCID mice [36]. Nevertheless, the exact molecular mechanisms behind this phenomenon remain uncertain.

The role of CD133 in cancer cells has been investigated from several perspectives. Experiments in normal and cancer cell lines have found that CD133 protein expression is higher in the G2/M phase than in the G1/G0 phase. This implies a relationship between CD133 protein expression and the cell cycle [37]. Hypoxia promotes CD133 expression in both stem cells and the tumor microenvironment, with increased expression of hypoxia-inducible factor (HIF)-1α in the background [38,39]. Observations also show that hypoxia-induced mitochondrial membrane potential alteration enhances CD133 protein expression through the post-transcriptional regulation of CD133 [39]. CD133 may alter the cytoskeleton, influencing glucose metabolism [40]. Furthermore, CD133 can inhibit transferrin uptake, which can affect oxygen transport under hypoxic conditions [41].

Given the diverse functions of CD133, mapping the signaling pathways that influence its expression is also warranted. In cancer stem cells and kidney cells, CD133 has been observed to induce Wnt/β-catenin signaling [40,42]. A decrease in CD133 expression induces the disappearance of β-catenin from the nucleus, as well as a decrease in canonical Wnt signaling [43]. HDAC6 (histone deacetylase 6) interacts with CD133 in mammalian cells [44]. HDAC6 causes stabilization of β-catenin. Inhibition of CD133 or HDAC6, however, enhances acetylation and degradation of β-catenin, which is proportional to the decrease in cell proliferation and tumorigenesis [44]. This highlights the role of CD133 as a potential therapeutic target. CD133 is an important regulator of the phosphoinositide 3-kinase (PI3K)/protein kinase B (AKT) signaling pathway in cancer cells, but the details of this are not yet fully understood [45]. However, despite its ubiquitous expression in both normal and transformed cells [40], the precise functional role of CD133 in cancer remains unclear.

## 3. Molecular Links between CD133 and Apoptosis

CD133 modulates the apoptotic process in CRC through a variety of molecular mechanisms that impact important communication pathways and regulatory proteins that control cell death.

### 3.1. PI3K/AKT Pathway

CD133+ cells often activate the PI3K/AKT pathway, which promotes cell survival and inhibits apoptosis [46]. The activation of AKT can result in the phosphorylation and inactivation of pro-apoptotic factors, including Bcl-2-associated death promoter (Bad) and caspase-9, thereby preventing apoptosis. This pathway also boosts cell survival by upregulating anti-apoptotic proteins such as B-cell lymphoma 2 (Bcl-2) and B-cell lymphoma-extra-large (Bcl-xL) [47,48]. During the initial phases of tumor formation, primary malignant cells endure in a low-nutrient environment that is the result of a poorly organized vascular system. CD133 prevents serum starvation-induced colon cancer cell mortality by activating Akt-mediated anti-apoptosis and protein synthesis pathways, as demonstrated by knockdown and overexpression experiments [49]. Additionally, PI3K at least partially regulated the increase in endogenous CD133 protein quantity following serum deprivation [47]. Consequently, it is highly probable that CD133 plays a role in the development and maintenance of resistance to nutrient deprivation-induced stress in early avascular tumor tissues.

The PI3K/AKT pathway also boosts the expression of ABC transporters (e.g., ATP binding cassette subfamily B member 1 /ABCB1/ or multidrug resistance 1 /MDR1/) containing p-glycoprotein (P-gp), which has an antitumor effect. This is because activating the transporter pathway can lower the response to chemotherapeutic drugs and increase drug efflux [46]. CD44+CD133+ HCT8 CRC cells were found to exhibit resistance to doxorubicin [50]. Studies have shown that low-dose cisplatin treatment of non-small cell lung cancer cells effectively concentrates CD133+ cells and upregulates ABCB1 expression via Notch signaling [51]. This, in turn, enhances the cross-resistance to doxorubicin. Even so, verapamil’s effect on ABCB1 was small, and it only made CD44+CD133+ cells less sensitive to doxorubicin [50]. While interference experiments fail to provide direct evidence, it is possible that pathways involved in stress protection and redox regulation may have played a role, to some extent, in the resistance of CD44+CD133+ cells to doxorubicin. Surprisingly, silencing RNA (siRNA) suppression of CD133 expression in CD44+CD133+ HCT8 cells only slightly increases doxorubicin’s cytotoxicity, leaving the expression of ABCB1 and the intracellular accumulation of doxorubicin unchanged [50]. The expression of CD133 has a stronger correlation with the resistance of HCT8 CRC cells to anticancer drugs compared to CD44. The CD44+CD133+ HCT8 cells exhibited increased ABCB1 expression and reduced intracellular levels of doxorubicin accumulation [50]. Given the intricate processes of drug excretion and redox regulation that are anticipated to contribute to the drug resistance of CRC CSCs, it may be necessary to employ several strategies to address the significant issue of drug resistance in CRC patients.

Researchers have also shown that increased expression of MDR1/P-gp is associated with increased CD133 expression using LoVo and HCT8 CRC cell lines and human samples [52]. CD133 affects MDR1 expression through the AKT/nuclear factor (NF)-κB/MDR1 pathway. These results suggest that targeting CD133 through the AKT/NF-κB/MDR1 pathway reverses drug resistance, and that this pathway may serve as a potential therapeutic target in CRC.

While investigating the effect of metformin on CRC stem cell apoptosis and proliferation via microRNAs (miRNAs), recent research demonstrated that metformin reduces the expression of CD133 mRNA in CD133+ SW480 and HCT116 CRC stem cells by increasing the levels of miR-342-3p in an environment with a high glucose concentration [53]. This analysis presents novel concepts for prospective management of individuals diagnosed with diabetes and colon cancer using metformin.

### 3.2. Notch Signaling

**Figure 2:**
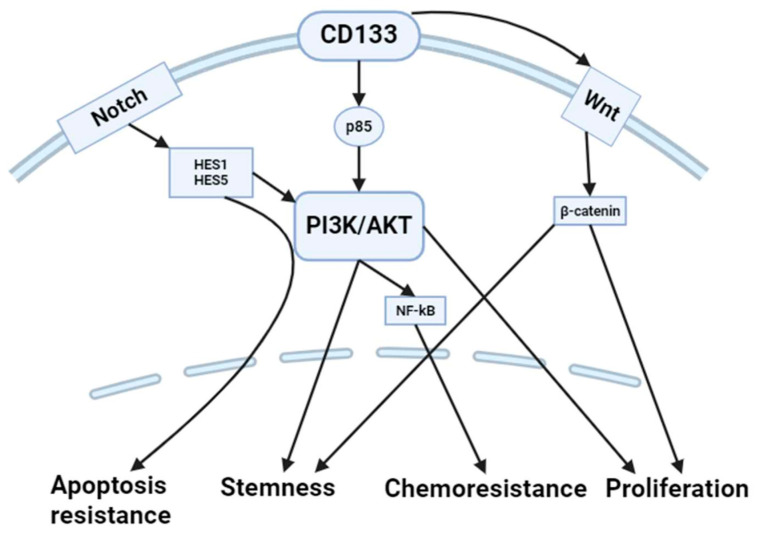
Several CD133 mechanisms have been suggested for CRC control. Notch activation effectively inhibits apoptosis by upregulating anti-apoptotic genes such as Hes1 and Hes5, as well as engaging with the PI3K/AKT pathway. The initiation of this signaling cascade allows CD133+ cells to evade apoptosis and preserve their stem-like properties. Activated CD133 physically interacts with WNT, resulting in elevated β-catenin levels. The β-catenin subsequently translocates to the nucleus, culminating in enhanced transcription of WNT target genes, which promotes stemness and proliferation of cancer cells. Activated CD133 phosphorylates the p85 subunit of PI3Ks. This cascade of molecular activations subsequently leads to the activation of AKT, which enhances stemness and proliferation. In addition, AKT turns on the NF-κB pathway, which increases the production of MDR-1 and makes cells chemoresistant. HES: hairy and enhancer of split; PI3K: phosphoinositide 3-kinase; AKT: protein kinase B; NF-kB: nuclear factor kappa B. The figure was partially created with https://www.biorender.com (accessed on 20 September 2024).

Cancer cells expressing CD133+ frequently display increased Notch signaling, a process that plays a role in preserving stemness and facilitating cell survival [54]. Notch activation effectively suppresses apoptosis by increasing the expression of anti-apoptotic genes such as hairy and enhancer of split (Hes)1 and Hes5 and by interacting with the PI3K/AKT pathway. The activation of this signaling cascade enables CD133+ cells to escape apoptosis and maintain their stem-like characteristics (Figure 2).

Studies have shown a positive correlation between the expression of Hes1 and the levels of stem cell markers, including CD133, ABCG2, ALDH1 (aldehyde dehydrogenase 1), Nanog (homeobox protein), Bmi-1 (B cell-specific Moloney-MLV Integration site 1), and CD44. This correlation suggests that Hes1 expression may increase the proliferation of stem-like CRC cells [55,56]. The Notch signaling pathway has been used to identify the impact of Hes1 on the preservation of specific stem cells and progenitor cells, as well as its partial influence on the digestive systems [57]. Specifically, the development of digestive organs involves interactions between Hes and Notch [57]. Furthermore, Hes1 serves as an indicator of healthy colon stem cells [58]. Nevertheless, an upregulation of Notch signaling, namely the Notch target Hes1, could potentially play a role in the manifestation of CRC [59].

It has been established that intra-tumoral heterogeneity of CSCs in CRC is a factor in chemoresistance and disease recurrence [60,61]. Even though slow-cycling colorectal CSCs are not very common, this permanent subpopulation may act as a more chemoresistant reserve population that is needed for recurrence and tumoral repopulation after treatment. NOTCH1 (NOTCH transmembrane mechanosensitive receptor 1) signaling through Hes1 and Hes5 modulation has been identified as the primary regulator of asymmetric division, which directly influences heterogeneity-based chemoresistance by regulating the balance between intra-tumoral CRC stem cell populations [60].

### 3.3. p53 Pathway

In response to DNA damage and cellular damage, the tumor suppressor protein p53 is essential in the induction of apoptosis. p53 has the ability to transcriptionally activate the expression of CD133 [62]. The HCT116 p53+/+ and p53−/− colon adenocarcinoma cell lines have been found to exhibit a substantial difference in the ratio of CD133-positive cell populations. The silencing or overexpression of p53 either reduced or restored CD133 expression. Informatics analysis predicted a 1313 bp fragment upstream of the CD133 transcriptional start site to contain putative p53-binding responsive elements. It was also proven by a dual-luciferase reporter assay that p53 effectively started transcription from the CD133 promoter region [62].

On the contrary, p53 expression levels increased and CD133 levels decreased in a dose-dependent manner following treatment with doxorubicin, a potent genotoxic agent [63]. The results show that higher levels of p53 caused by UV light or Nutlin-3, a substance that inhibits MDM2 (mouse double minute 2 homolog) from binding to p53 and stabilizes it, decreased the levels of CD133 in NTERA2 and NCCIT embryonic epithelial-like cells [63]. Doxorubicin, UV light, or Nutlin-3, however, increased the expression of p53-positive target genes such as *p21* or *p53 upregulated modulator of apoptosis* (*PUMA*) and decreased the expression of Nanog, which is a p53-repressive target gene [63].

The flavonoid quercetin induces apoptosis by activating caspase-9 and caspase-3, pro-apoptotic Bax upregulation, anti-apoptotic Bcl-2 post-translational modification, and DNA fragmentation [64]. In CD133+ HT29 colon adenocarcinoma cells, the administration of quercetin enhances the anticancer effects of doxorubicin and induces cell cycle arrest and apoptosis [64].

A recent study has shown that the impaired sensitivity of mutant p53-harboring HT29 colon cancer cells to oxaliplatin is significantly influenced by the dysregulated phosphorylation of the N- and C-terminus serine residues of the mutant p53 protein [65]. The impaired sensitivity of mutant p53-harboring colon cancer cells to oxaliplatin is also hypothesized to be influenced by cancer stemness (represented by CD133 and CD44 positivity) and Wnt/β-catenin signaling [65].

In conclusion, the molecular connections illustrate how CD133+ colorectal cancer cells enhance their survival and maintain their stem cell-like characteristics by inhibiting critical apoptotic pathways, thereby rendering them resistant to conventional treatments. A thorough comprehension of these processes provides valuable insights for the creation of highly targeted therapies that specifically target the induction of apoptosis in CD133+ cancer cells.

## 4. Interconnection of CD133 and Autophagy

Many important biological processes and regulatory mechanisms mediate the interaction between CD133 and autophagy in colorectal cancer cells. This can lead to enhanced tumor growth, resistance to apoptosis, and increased capacity for self-renewal.

### 4.1. PI3K/AKT/mTOR Pathway

The PI3K/AKT/mechanistic target of rapamycin (mTOR) signaling pathway, in which CD133+ cells frequently display abnormalities, plays a critical role in regulating autophagy [66]. This route can suppress autophagy by activating mTOR, which acts as a negative regulator of autophagy onset [67]. However, in the context of CD133+ cancer stem cells, this specific pathway can be manipulated to maintain a delicate balance between cell survival and autophagy, depending on the unique biological conditions and external influences.

**Figure 3:**
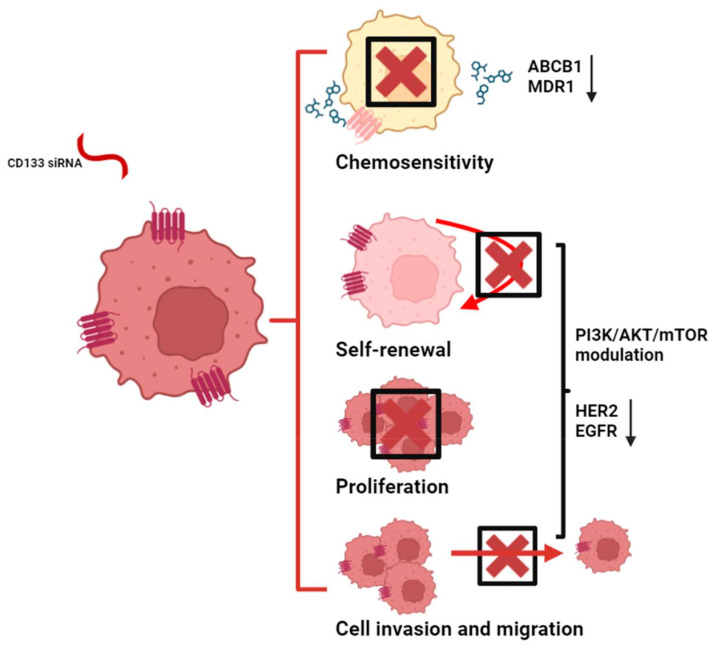
Inhibiting CD133 function in colorectal cancer stem cells (e.g., through CD133 silencing RNAs) may enhance cancer stem cell chemosensitivity by decreasing ABCB1 or MDR1 expression. Additionally, it attenuates the self-renewal potential of cancer stem cells, as well as cell proliferation, invasion, and migration, by modifying the PI3K/AKT/mTOR pathway and diminishing HER2 expression and EGFR activity. siRNA: silencing ribonucleic acid; ABCB1: ATP binding cassette subfamily B member 1; MDR1: multidrug resistance 1; PI3K: phosphoinositide 3-kinase; AKT: protein kinase B; mTOR: mechanistic target of rapamycin; HER2: human epidermal growth factor receptor 2; EGFR: epidermal growth factor receptor. The figure was partially created with https://www.biorender.com (accessed on 20 September 2024).

CD133 has been shown to enhance the activation of key survival and proliferation pathways, including the PI3K/AKT/mTOR axis [68]. Apart from its capacity to recruit p85 and promote subsequent Akt activation, CD133 can also regulate Akt activation by stabilizing receptor tyrosine kinases [69]. CD133, for example, confers stability to epidermal growth factor receptor (EGFR) in the plasma membrane via direct contact [70,71]. CD133 evades EGFR internalization and maintains EGFR-induced Akt activation [70,71]. In CRC cell lines, the suppression of CD133 reduces the expression of HER3 (human epidermal growth factor receptor 3) and inhibits the activation of EGFR and HER2 (human epidermal growth factor receptor 2) without disrupting their translation [72]. In CRC, CD133 silencing partially inhibits proliferation, clonogenic capacity, migration, cell invasion, and resistance by reducing glucose uptake due to reduced glucose transporter 1 expression [72,73] (Figure 3). This is caused by an impaired HER3/Akt pathway, which is involved in protein synthesis via mTOR and mRNA stability. It is likely that this effect is mediated by the regulation of RNA-binding proteins [74].

Upregulation of the PI3K pathway is frequently observed in CRC [75,76]. Although CD133 does not directly stimulate PI3K, CD133+ cells often have elevated levels of PI3K activity [69]. This phenomenon may be attributed to the interactions between CD133+ CSCs and other growth factors or surface receptors that activate PI3K signaling pathways [77,78].

Once PI3K is activated, it induces the phosphorylation and activation of AKT. CD133-positive CSCs frequently exhibit elevated AKT phosphorylation, which is pivotal in the activation of processes such as cell proliferation, survival, autophagy, and resistance to therapy, including chemotherapy [52,79,80].

The PI3K/AKT/mTOR pathway in CD133-positive cells contributes to their enhanced capacity for survival and resistance to environmental stress, such as nutrient deprivation or hypoxia, which is common in tumor microenvironments [81]. Multiple studies have demonstrated that CD133 can activate the PI3K/AKT signaling pathway in several cancer cells [82]. Autophagy was shown to be a critical factor in the regulation of drug resistance by CD133, and the activation of the mTOR signaling pathway resulted in a decrease in autophagy and an improvement in drug therapeutic efficacy [83,84]. Similarly, in CD133+ cisplatin-resistant gastric cancer cells, co-incubation with sh-CD133 and cisplatin significantly inhibited the PI3K/AKT/mTOR signaling pathway [81]. The PI3K/AKT/mTOR signaling pathway may have been a key part of CD133-mediated cisplatin resistance. Consequently, the activation of the PI3K/AKT/mTOR signaling pathway by CD133 may enhance cisplatin resistance in the tumor microenvironment.

In conclusion, CD133 plays a crucial role in the increased activation of the PI3K/AKT/mTOR pathway in cancer cells, thereby contributing to their aggressive behavior and resistance to therapies. Despite the fact that it does not directly activate the pathway, it is closely related to it.

### 4.2. AMPK Pathway

CD133+ cells can also influence autophagy through the adenosine monophosphate (AMP)-activated protein kinase (AMPK) pathway, which senses cellular energy status. Compared to non-CRC cancer stem cells, CRC CSCs showed reduced levels of reactive oxygen species and elevated levels of antioxidant genes. The CRC CSCs also have a higher mitochondrial mass and above-average mitochondrial activity. More notably, increased AMPK activities were detected in CRC CSCs [85]. AMPK activation can promote autophagy by inhibiting mTOR and activating ULK1 (Unc-51 like autophagy activating kinase 1), a key initiator of the autophagic process [86,87]. This pathway helps maintain cellular energy homeostasis under metabolic stress conditions, supporting the survival of CD133+ cancer cells.

The functional study of the CD133 gene in mice found that knocking out *Prom1* enhanced AMPK activation, reduced autophagy, and elevated the expression of epithelial–mesenchymal transition (EMT) signals in mouse retinal pigment epithelial (RPE) cells, indicating a potential association between autophagy inhibition and EMT [88]. In accordance with these findings, the stimulation of autophagy has demonstrated the ability to counteract EMT and preserve RPE homeostasis [89].

### 4.3. Hypoxia-Inducible Factors

Most solid tumors, including colon cancer, exhibit rapid cell growth in central hypoxic areas due to their limited blood supply. The hypoxic microenvironment and the activation of its downstream pathways link to the pathogenesis and metastasis of cancer [90].

In the tumor microenvironment, hypoxia can induce the expression of HIFs, which are known to regulate autophagy [90]. CD133+ cells, which thrive in hypoxic niches, may utilize HIF-mediated autophagy to adapt to low oxygen conditions and promote their survival and stemness properties [91]. HIFs can upregulate autophagy-related genes and enhance the autophagic flux, providing a survival advantage to CD133+ cancer stem cells under hypoxic stress [92].

The mRNA and protein expressions of CD133 and ezrin (EZR), a member of the Ezrin–Radoxin–Moesin (ERM) family, which acts as an intermediary between the plasma membrane and the actin cytoskeleton, showed a highly significant positive correlation in CRC samples [93,94]. Recent research has shown that hypoxia-induced autophagy is responsible for the initiation and progression of colorectal cancer by activating the protein kinase C-EZR pathway [95].

### 4.4. Beclin-1 and Bcl-2 Interactions

Beclin-1 is a crucial autophagy regulator that forms complexes necessary for autophagosome formation. Similar to a scaffold protein, Beclin-1 is capable of undergoing modifications throughout the entirety of the autophagy process. It is possible for c-Jun N-terminal protein kinase 1 (JNK1) to directly phosphorylate Bcl-2 in conditions of hunger, which results in the release of free Beclin-1 from the Bcl-2/Beclin-1 complex and, thus, the regulation of autophagy [96]. Through the process of phosphorylating Beclin-1, a wide variety of different kinases, including AMPK, Akt, EGFR, and MK2 (MAP KAP kinase 2), have the potential to regulate the Beclin-1-Vps34 complex [97]. In light of the fact that CD133 is capable of interacting with all of JNK, AMPK, Akt, or EGFR [98], it is expected that CD133+ cells have the ability to control the interaction between Beclin-1 and Bcl-2. This regulatory mechanism highlights the interplay between autophagy and apoptosis in maintaining the viability of CD133+ CSCs.

### 4.5. Transcriptional Regulation

Through the transcriptional regulation of autophagy-related genes, CD133+ cells can influence autophagy. Transcription factors, such as forkhead box O (FOXO)3a, can influence the expression of genes involved in autophagy. Inhibition of FOXO3a has been shown to significantly enhance cancer stem cell properties in lung cancer. It enhances the expression of stem cell markers (i.e., SOX2 /SRY-Box Transcription Factor 2/, Nanog, KLF4 /Krüppel-like factor 4/, ALDH, and CD133) as well as spheroid formation. In addition, it reduces the level of the proapoptotic effector protein BIM (Bcl-2 interacting mediator of cell death) [99]. By utilizing these transcriptional networks to fine-tune autophagy and adapt to a variety of stressors, CD133+ cells may improve their survival and resistance to therapies. Nevertheless, there is no direct evidence in the literature of how CD133 influences autophagy through FOXO3a in CRC.

In general, CD133+ CSCs employ autophagy as a survival mechanism during cancer therapy or in response to metabolic duress. FOXO3a is a member of the forkhead transcription factor family’s subgroup O and exerts a significant influence on the aging process by modulating a variety of life processes, such as cell cycle arrest, apoptosis, autophagy, oxidative stress, and DNA repair [100].

CD133 and FOXO3a may not directly interact, but they may both be involved in the same cellular survival pathways. It is evident from the aforementioned that CD133+ cells frequently possess an activated PI3K/AKT signaling pathway, which can obstruct FOXO3a’s nuclear translocation [101]. FOXO3a inhibition can decrease its capacity to activate genes associated with autophagy, indicating a possible indirect coupling [102].

In addition, CD133+ cancer cells can modulate the mTOR pathway, which controls both FOXO3a activity and the autophagy programs [103,104]. AMPK directly stimulates autophagy by phosphorylating autophagy-related proteins within the mTORC1 (mammalian target of rapamycin complex 1), ULK1, and PIK3C3/Vps34 complexes, or indirectly by modulating the expression of autophagy-related genes regulated by transcription factors such as FOXO3, TFEB (transcription factor EB), and BRD4 (bromodomain protein 4) [105]. In lung cancers, AMPK may employ distinct methods to affect the transcriptional ability of FOXO3. In small cell lung cancer, phosphorylated AMPK lowers the phosphorylation of FOXO3a. This decreases the expression of cancer stem cell markers like CD133 [106]. On the other hand, in breast cancer stem cells and prostate cancer cells, the downregulation of FOXO3A leads to an increase in the expression of CD133 on the cell membrane [107,108].

FOXO3a controls genes that safeguard cells against oxidative stress, and autophagy is a cellular process employed to eliminate degraded proteins and organelles resulting from oxidative damage [109]. Enhanced autophagic flux may enable CD133+ cells to effectively regulate oxidative stress [110,111,112]. FOXO3a was identified as a potential transcription factor implicated in the modulation of these responses [113,114].

HIFs have a crucial role in controlling both autophagy and stem cell-like properties in cancer cells. HIFs can regulate FOXO3a, and hypoxia often triggers an increase in CD133 expression [115,116]. Therefore, within a tumor microenvironment characterized by low oxygen levels, CD133 and FOXO3a may cooperate in a common regulatory network.

In fact, there is no solid evidence that links CD133 to FOXO3a in CRC. However, it is possible that the two may work together through common signaling pathways like PI3K/AKT and mTOR, or in response to oxidative stress and hypoxia. Autophagy may indirectly influence the survival capacity of CD133+ cells through transcriptional modifications involving FOXO3a expression. Further study of these pathways may reveal more information about their indirect interactions.

## 5. Interconnection of CD133-Mediated Autophagy and Apoptosis in CRC

The previous chapters have shown that CD133+ cells in CRC frequently display increased autophagy, which allows them to avoid apoptosis. In order to prevent stress-induced apoptosis, autophagy enables CSCs to eliminate damaged organelles and proteins, therefore preserving cell homeostasis. Autophagy recycles intracellular components to supply vital nutrients and energy to CSCs, allowing CD133+ cells to thrive in situations with limited nutrient availability.

Direct experiments on CRC have shown that blocking autophagy in CD133+ cancer cells makes them more likely to die through activated cell death. When used with chemotherapeutic drugs like 5-fluorouracil (5-FU) and oxaliplatin, stopping the activity of key autophagy-related genes (like *autophagy protein 5* /*ATG5*/ or *Beclin-1*) in CD133+ CRC cells raises the death rate [117,118].

In CRC models, chloroquine and hydroxychloroquine modulate CD133+ cell death by suppressing the autophagy process [112]. The inhibition of autophagy by these medications may induce apoptosis in CSCs that would otherwise exhibit resistance to chemotherapy.

Furthermore, chemotherapy or radiation can induce autophagy and apoptosis. CD133+ cells frequently exhibit a preference for autophagy as a means to evade apoptotic demise. Following treatment, CD133+ cancer cells increase the expression of stress-induced autophagy, which facilitates the restoration of impaired cellular components and hence decreases the probability of apoptosis [119]. This is especially important for cancer cells experiencing oxidative damage, hypoxia, or nutritional deprivation.

CD133+ cancer cells frequently exhibit increased expression of Bcl-2 family members, including Bcl-xL and Mcl-1 (induced myeloid leukemia cell differentiation protein), which serve to prevent apoptosis by stabilizing the mitochondrial membrane [120,121]. Suppression of autophagy can disturb this equilibrium and stimulate mitochondrial-mediated apoptosis, a process commonly seen in CRC [122,123,124].

CD133+ cells in CRC frequently exhibit EMT, a mechanism that facilitates the spread of cancer cells to distant sites and confers resistance to cell death [125]. Enhanced EMT is also associated with increased autophagy, which helps cancer stem cells survive and invade [126]. By suppressing autophagy in EMT-like CD133+ cells, it is possible to reinstate their responsiveness to apoptosis, therefore decreasing the occurrence of metastasis and inhibiting tumor advancement [127].

Analysis of CD133+ CRC cells revealed that CD133+ cells exhibited more autophagic activity in comparison to CD133- cells [128]. When exposed to autophagy inhibitors, the CD133+ cells exhibited heightened apoptosis and decreased survival, especially in the presence of chemotherapy-induced stress [129,130]. This emphasizes the defensive role of autophagy in preventing apoptosis in CD133+ cells. Furthermore, the knockdown of *ATG* genes, or inhibiting autophagy in CD133+ cells using xenograft CRC models, resulted in heightened apoptosis and decreased tumor growth [131]. This finding also supports the idea that autophagy plays a crucial role in CD133+ cells avoiding apoptosis and resisting treatments.

**Figure 4:**
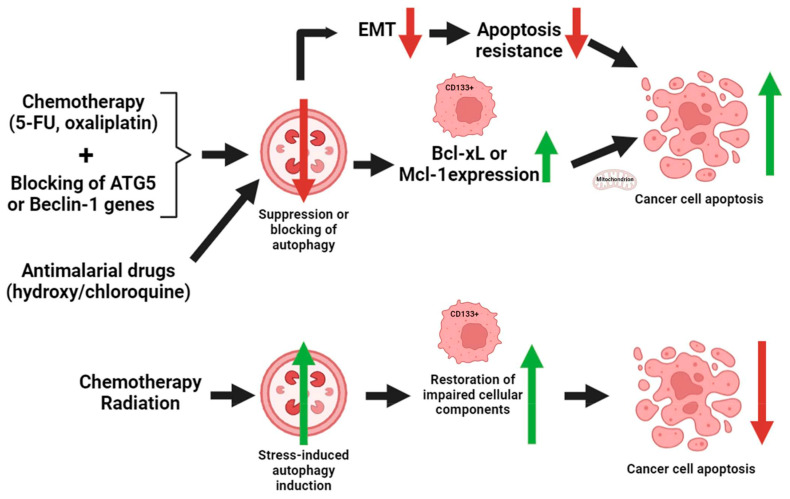
Correlation of autophagy and apoptosis with CD133 in colorectal cancer. Suppressing autophagy through the attenuation of epithelial–mesenchymal transition diminishes apoptosis resistance, ultimately leading to the demise of cancer cells. Inhibition of autophagy reduces the expression of anti-apoptotic proteins in CD133+ cancer cells, resulting in death due to mitochondrial membrane instability. Chemotherapeutic medicines, autophagy gene suppression, or antimalarial medications can reduce or block the autophagy process. Chemotherapy or irradiation may simultaneously decrease apoptosis by promoting stress-induced autophagy, which facilitates the repair of damaged cellular components. EMT: epithelial–mesenchymal transition; 5-FU: 5-fluorouracil; ATG5: autophagy protein 5; Bcl-xL: B-cell lymphoma-extra-large; Mcl-1: myeloid cell leukemia-1; red arrows: decrease; green arrows: increase. The figure was partially created with https://www.biorender.com (accessed on 9 October 2024).

In conclusion, CD133+ cells in CRC frequently depend on autophagy to endure adverse environments, including cancer treatments. Autophagy assists these cells in evading apoptosis by preserving cellular homeostasis, fulfilling metabolic requirements, and alleviating therapy-induced damage. Inhibiting autophagy in CD133+ cells has demonstrated increased susceptibility to apoptosis, presenting a possible therapeutic method to address treatment resistance (Figure 4).

## 6. Therapeutic Considerations

The therapeutic potential of CD133 as a target in CRC is substantial, as it is associated with CSCs and plays a role in evading apoptosis and utilizing autophagy for survival. CD133-positive cells are a prospective yet challenging target for therapy due to their distinctive characteristics.

The targeting of CD133+ CSCs is a beneficial approach [132]. Antibodies that target CD133 have the potential to directly bind and eradicate CD133+ cells, thereby indicating them for immune-mediated clearance. Some preclinical investigations have focused on anti-CD133 monoclonal antibodies that either conjugate to cytotoxic agents (antibody-drug conjugates or nanoparticle-based delivery systems) or block CD133 function. This method involves directly delivering a toxic payload to CD133+ cells by attaching a cytotoxic substance to an antibody that specifically targets CD133. This approach specifically eliminates CSCs, thereby diminishing systemic toxicity. Chimeric antigen receptor (CAR) T cells that are engineered to target CD133 are currently being investigated as a method of selectively killing CSCs. These CAR T cells are engineered to bind to CD133 and trigger a robust immune response, thereby eliminating CSCs that contribute to cancer relapse and metastasis [132,133,134].

Additionally, the inhibition of autophagy in CD133+ cancer cells is a significant alternative [135]. The combination of traditional cancer therapies with autophagy inhibitors (e.g., chloroquine or hydroxychloroquine) could potentially increase the susceptibility of CD133+ cells to treatment, as they frequently depend on autophagy for survival under duress. The inhibition of the autophagic pathway may compromise the metabolic resilience of CSCs. It is also possible to increase the death rate of CD133+ CRC cells by combining autophagy inhibitors with pro-apoptotic agents. Disrupting both autophagy and apoptosis resistance mechanisms may achieve the potential to overcome treatment resistance [135,136].

Cardiac glycosides, such as neriifolin, oleandrin, or bufalin, target Beclin-1 and inhibit the production of LC3-associated phagosomes [137,138,139]. In CRC, bufalin induces cell death by increasing reactive oxygen species formation and inducing autophagy without signs of apoptosis [139]. Moreover, cardiac glycosides can reduce cancer cell stemness (in part, i.e., the expression of CD133) via inhibiting HIF-1α [140]. Their anticancer mechanism appears to be complex and not entirely elucidated; yet, autophagy has been established as a crucial factor in this process. The molecular mechanisms involved in this process highlight their potential for use in clinical practice.

Another beneficial treatment option is to induce apoptosis in CD133+ CRC cells [141,142,143]. CD133+ CSCs frequently upregulate anti-apoptotic proteins like Bcl-2 or Mcl-1. Small-molecule inhibitors, such as BH3 (B-cell lymphoma 2 homology 3) mimetics, that target these anti-apoptotic proteins could restore the apoptotic sensitivity of CD133+ cells [144]. This combination of agents with chemotherapy has the potential to improve the overall therapeutic effect. RNA interference or clustered regularly interspaced short palindromic repeats (CRISPR)-based gene-editing technologies could silence the expression of CD133 in CRC cells [145,146]. Re-sensitizing CSCs to apoptosis by reducing CD133 expression may improve the efficacy of standard therapies.

Moreover, the integration of immunotherapies with CD133 targeting appears to be a promising alternative. Combining therapies that target CD133 with immune checkpoint inhibitors (e.g., anti-PD-1 /programmed cell death protein 1/ or anti-CTLA-4 /cytotoxic T-lymphocyte associated protein 4/) may overcome the immune evasion strategies employed by CD133+ CSCs [147]. This combination therapy has the potential to provide a strong anti-tumor response by simultaneously reducing CSC resistance and stimulating the immune system. It may be possible to develop CD133-targeted vaccines that specifically target CD133+ CRC cells to trigger an immune response [148]. This method could potentially prevent recurrence by priming the immune system to identify and combat CSCs.

Small-molecule inhibitors and agonists/activators of CD133-interacting proteins are also potential therapeutic options.

Amcasertib (BBI503) is an orally bioavailable small-molecule multi-kinase inhibitor [149]. In PC-9/GR lung adenocarcinoma cells, amcasertib inhibits the expression of Nanog and CD133 and also blocks the cells from dividing. It is an orally bioavailable kinase inhibitor targeting cancer cell stemness, exhibiting a potential antineoplastic effect, and is presently undergoing phase I clinical trials across various malignancies [149].

A notable decrease in tumor development derived from CD133+ tumor-initiating cells was accomplished, both in vitro and in vivo, through specific AKT inhibition with MK-2206. AKT serves as both an upstream activator of mTOR complex 1 and a downstream effector, with its overexpression linked to chemotherapy resistance, making it a pivotal target within this signaling cascade. Consequently, selective AKT inhibition may surpass mTOR inhibitors in the management of CRC and serves as a potential strategy to avert tumor recurrence by targeting cancer stem cells [150].

**Figure 5:**
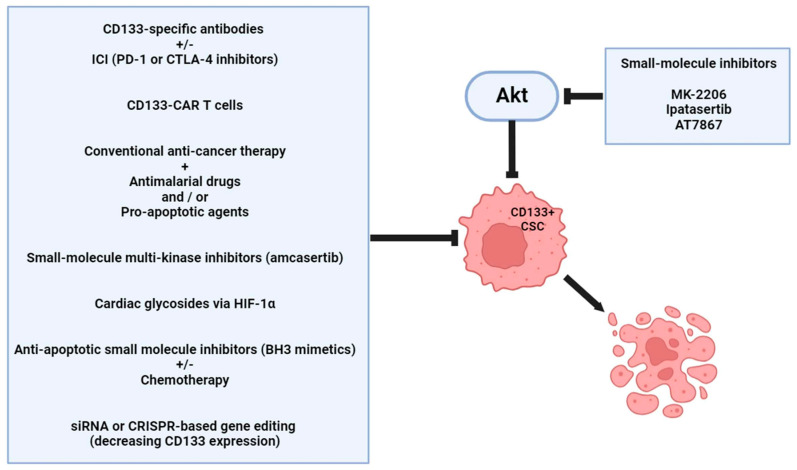
Targeted therapeutic options for CD133 and related molecules represent a new approach to the treatment of colorectal cancer. ICI: immune checkpoint inhibitor; PD-1: programmed cell death protein 1; CTLA-4: cytotoxic T-lymphocyte associated protein 4; CAR T: chimeric antigen receptor T cell; HIF-1α: hypoxia-inducible factor 1α; BH3: B-cell lymphoma 2 Homology 3; siRNA: small interfering RNA; CRISPR: clustered regularly interspaced short palindromic repeats; CSC: cancer stem cell. The figure was partially created with https://www.biorender.com (accessed on 9 October 2024).

Ipatasertib is a highly selective, orally bioavailable inhibitor of Akt kinase [151]. Ipatasertib promoted apoptosis in colon cancer cells by activating PUMA, a process reliant on FOXO3a and NF-κB but independent of p53. PUMA was necessary alone for ipatasertib therapy and in combination with additional drugs for colon cancer treatment [151]. The AT7867 Akt inhibitor was also identified as a suppressor of colorectal cancer-derived stem cells via modulating the stem cell maintenance factor Ascl2 and Akt signaling pathways [152] (Figure 5).

## 7. Future Directions

In order to optimize therapeutic strategies, additional investigation into the precise biological function of CD133, particularly in CRCs, is required. CD133 is not merely a marker; it may also modulate cellular pathways that are essential for the survival and maintenance of CSCs. The considerable heterogeneity of colorectal tumors suggests that targeting CD133 alone may not be sufficient. Understanding the manner in which CD133-positive cells interact with other components of the tumor microenvironment will facilitate the development of more comprehensive treatments. Patient-specific therapies that target CD133 and its interacting proteins could be developed as our understanding of CD133 and its function in CRC increases, thereby guaranteeing that treatments are customized to the distinctive molecular profile of each individual’s tumor.

## Data Availability

No new data were created.
